# Social Prescribing Outcomes for Trials (SPOT): Protocol for a modified Delphi study on core outcomes

**DOI:** 10.1371/journal.pone.0285182

**Published:** 2023-05-16

**Authors:** Elham Esfandiari, Anna M. Chudyk, Sanya Grover, Erica Y. Lau, Christiane Hoppmann, W. Ben Mortenson, Kate Mulligan, Christie Newton, Theresa Pauly, Beverley Pitman, Kathy L. Rush, Brodie M. Sakakibara, Bobbi Symes, Sian Tsuei, Robert J. Petrella, Maureen C. Ashe

**Affiliations:** 1 Department of Family Practice, The University of British Columbia (UBC), Vancouver, British Columbia (BC), Canada; 2 College of Nursing, Rady Faculty of Health Sciences, University of Manitoba, Winnipeg, Manitoba, Canada; 3 Department of Emergency Medicine, UBC, Vancouver, BC, Canada; 4 UBC Department of Psychology, UBC, Vancouver, BC, Canada; 5 UBC Department of Occupational Science and Occupational Therapy, Vancouver, BC, Canada; 6 GF Strong Rehabilitation Research Program, Vancouver, BC, Canada; 7 International Collaboration on Repair Discoveries, Vancouver, BC, Canada; 8 Dalla Lana School of Public Health, University of Toronto, Ontario, Canada; 9 Department of Gerontology, Simon Fraser University, Burnaby, BC, Canada; 10 United Way British Columbia, Burnaby, BC, Canada; 11 School of Nursing, UBC-Okanagan, Kelowna, BC, Canada; 12 Centre for Chronic Disease Prevention and Management, Southern Medical Program, UBC-Okanagan, Kelowna, BC, Canada; 13 Department of Global Health and Population, Harvard T H Chan School of Public Health, Boston, MA, United States of America; 14 Department of Family Medicine, Western University, London, Ontario, Canada; 15 School of Kinesiology, Western University, London, Ontario, Canada; 16 Western Centre for Public Health & Family Medicine, Western University, London, Ontario, Canada; PLoS ONE, UNITED STATES

## Abstract

**Purpose:**

This is a study protocol to co-create with knowledge users a core outcome set focused on middle-aged and older adults (40 years+) for use in social prescribing research.

**Methods:**

We will follow the Core Outcome Measures in Effectiveness Trials (COMET) guide and use modified Delphi methods, including collating outcomes reported in social prescribing publications, online surveys, and discussion with our team to finalize the core outcome set. We intentionally center this work on people who deliver and receive social prescribing and include methods to evaluate collaboration. Our three-part process includes: (1) identifying published systematic reviews on social prescribing for adults to extract reported outcomes; and (2) up to three rounds of online surveys to rate the importance of outcomes for social prescribing. For this part, we will invite people (n = 240) who represent the population experienced in social prescribing, including researchers, members of social prescribing organizations, and people who receive social prescribing and their caregivers. Finally, we will (3) convene a virtual team meeting to discuss and rank the findings and finalize the core outcome set and our knowledge mobilization plan.

**Conclusion:**

To our knowledge, this is the first study designed to use a modified Delphi method to co-create core outcomes for social prescribing. Development of a core outcome set contributes to improved knowledge synthesis via consistency in measures and terminology. We aim to develop guidance for future research, and specifically on the use of core outcomes for social prescribing at the person/patient, provider, program, and societal-level.

## Introduction

Social prescribing is a model of health and social care which focuses on aspects of people’s unmet social needs [1]. The approach connects people with public and non-profit or volunteer community resources, such as physical activity, arts-based, or housing resources [2]. It aims to address, for example, loneliness or management of long-term health conditions, like diabetes [3]. This is an especially salient issue because of the COVID-19 pandemic and subsequent social isolation, which may have undermined the health and well-being of people across the life span [4].

For too long, health has been defined as the absence of disease and is less aligned with the World Health Organization’s definition as “*a state of complete physical*, *mental*, *and social well-being*, *and not merely the absence of disease…”* [5], or an updated version proposed as “*structural*, *functional and emotional state that is compatible with effective life as an individual and as a member of society*.*”* [6]. In the past several decades, a new health and social model of care, social prescribing, has emerged. The United Kingdom (UK)’s public healthcare system is leading the way with social prescribing with an objective to refocus the healthcare system on non-medical concerns [1]. While globally there is no accepted definition for social prescribing, a recent pre-print used a Delphi method to report social prescribing as “*a means for trusted individuals in clinical and community settings to identify that a person has non-medical*, *health-related social needs and to subsequently connect them to non-clinical supports and services within the community by co-producing a social prescription…”* [7].

There are a number of systematic reviews on social prescribing and its effect on health and well-being [2, 8–16]. Although results to date are inconclusive, there are some promising findings. However, there are several considerations for future research. For example, our systematic review of social prescribing for older adults [2], noted considerable heterogeneity in outcomes used among studies. Second, synthesizing evidence on social prescribing requires knowledge on outcomes at multiple levels of person, provider, and system. Systematically identifying and synthesizing patient (person)-important outcomes [17] for social prescribing is essential for research and practice. In particular, a focus on “patient-important” outcomes helps to better understand people’s preferences and values [18] and improve patient relevance by contributing to a consistent understanding [17]. Furthermore, evidence lacks sufficient detail about social prescribing implementation to be assessed for their effectiveness and value [8]. Trials sometimes need system-level considerations to explore factors that go beyond the person-level, for example, effective access, efficient resource utilization [19], and implementation outcomes at the system level [8]. These knowledge gaps (and possibly others) make it difficult to synthesize evidence on social prescribing outcomes at multiple levels (person, provider, system).

The Core Outcome Measures in Effectiveness Trials (COMET) is a research initiative to guide the identification and selection of measures [20] defined as “*an agreed standardized collection of outcomes … which should be measured and reported in all trials for a specific clinical area*” (p.1) [21]. Sets of core outcomes have been developed for several health conditions [22–27], and may reduce outcome reporting bias, enhance comparability and enable syntheses of results across different studies [21]. Core outcome sets can also provide a list of meaningful measures across relevant groups, such as patients/people, caregivers, healthcare providers, and/or policy makers, who affect or are affected by an intervention [28], which ultimately could improve care decisions [29, 30]. There are several approaches or methods used in the development of core outcome sets, such as conducting systematic reviews (to identify outcomes used in practice or research), and to reach consensus on core outcome measures, using methods such as the Delphi method [20, 31].

The Delphi method is frequently used in healthcare settings to reach consensus for decision-making [32–37]. It usually involves an iterative process with systematic rounds of (online) rating/ranking (of questions/concepts) to reach group agreement, and it is especially useful when there are few data available [34], which is the case for social prescribing. It is likely multiple outcomes will not reach agreement [23], therefore a modified version of the Delphi method may be more effective in the synthesis process [34, 38, 39]. Many modified approaches request participants first respond to survey questions (individually), followed by a group discussion to finalize the results [39]. Further, to increase the generalizability of the core outcomes set, it has been recommended to involve a variety of knowledge users in the surveys and group discussion [40]. Therefore, a modified Delphi method will provide opportunities to resolve potential disagreements between different knowledge users and ensure that the population with experience in social prescribing are represented in the process [23].

It is rare in practice for knowledge users, such as patients, to contribute early to the development of core outcomes sets [40]. Incorporating different knowledge users’ perspectives (especially patients) on outcomes is important for health care resource utilization [29, 41]. Patients as research team members are not “*just patients*” in the traditional sense [42]. In addition to their health conditions and needs, they have a wide variety of backgrounds, skills, and interests to be incorporated through an intersectional approach that acknowledges the relationship between different social locations and experiences [42, 43]. Participatory action research is a collaborative approach to engaging knowledge users, who can contribute to the resolution of a problem and benefit from the solution in a “*real-world setting*” [44, 45]. Core outcome set development could be strengthened with a participatory approach to support identification and consensus building for important outcomes, and in particular, for patient-important outcomes.

To advance the science and practice of social prescribing, a logical next step is to co-create a core outcome set. To our knowledge, there has not been a core outcome set developed for social prescribing, but there are two projects on core outcomes in social care [46, 47]. Specifically, there is one report of an umbrella review (review of reviews) on integrated health and social care for people living with chronic health conditions [46]; and the second project is the development of core outcomes for people receiving social care, related to the delivery of specific services such as home care, day centres, equipment, or home adaptations [47]. Our objective is to develop core outcomes for adults aged 40 years and older, when the perception of aging starts [48, 49], using a three-part modified Delphi method. We anticipate the core outcome set could be used in planning and testing future social prescribing research studies [32–34, 38, 39]. We further aim to extend Delphi methods by using a participatory approach [44, 45] and collaborate with a diverse group of people before (in team composition), during (recruiting participants) and after (within our knowledge mobilization strategies) the research study [50]. Therefore, the purpose of this work is to report on the protocol that will be used to guide our work of co-creating a core outcome set with knowledge users focused on middle-aged and older adults (40 years+) for use in social prescribing research.

## Materials and methods

We will use a three-part modified Delphi method [32–34, 38, 39, 51] to identify social prescribing core outcomes for middle-aged and older adults (40 years and older) at the person, provider, program, and societal-levels.

**Fig 1:**
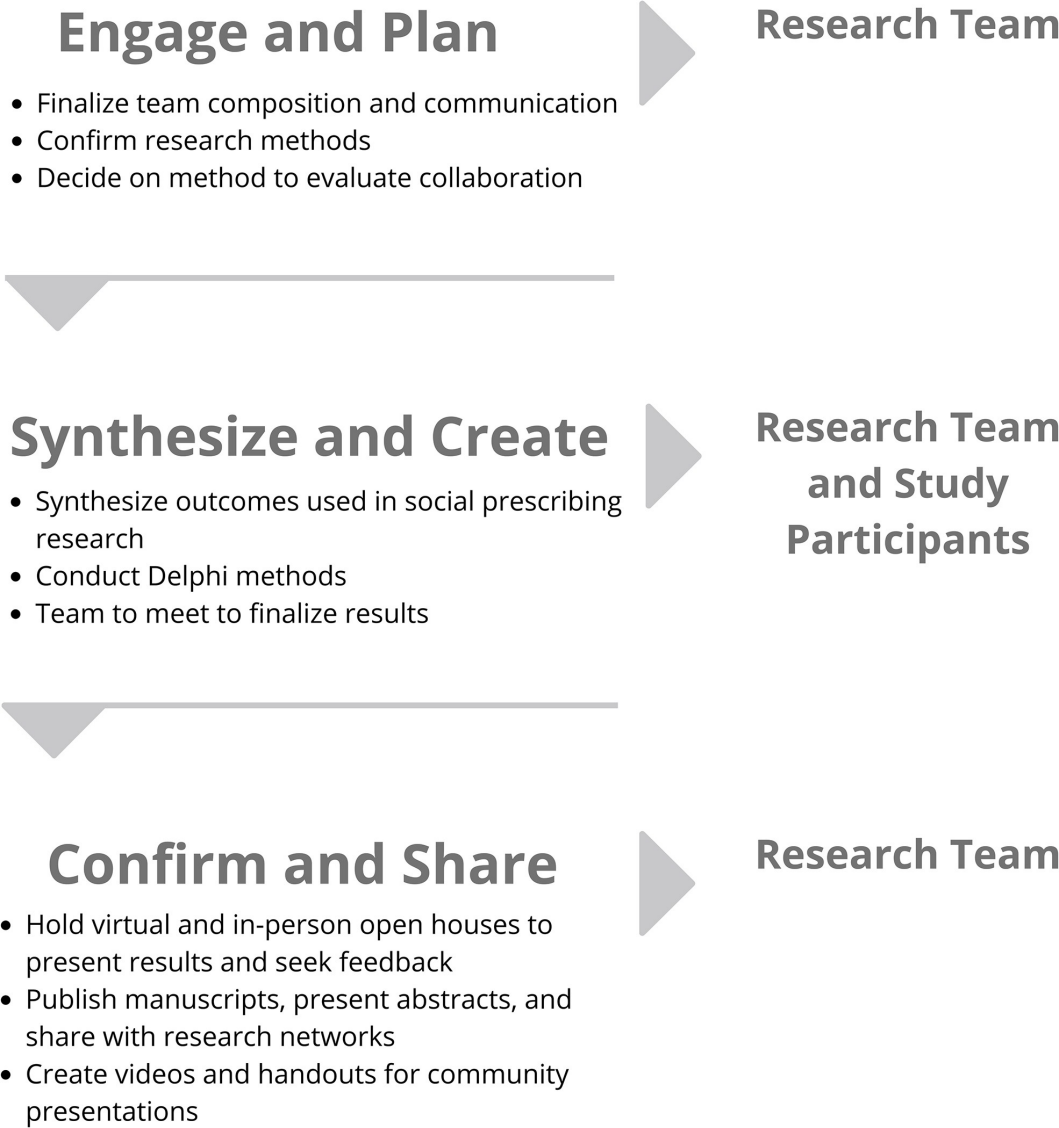
An overview of the co-creation, consensus, and communication process within this proposal. The figure outlines the steps within the process (the “what”) and people involved with each step (“the who”).

**Fig 2:**
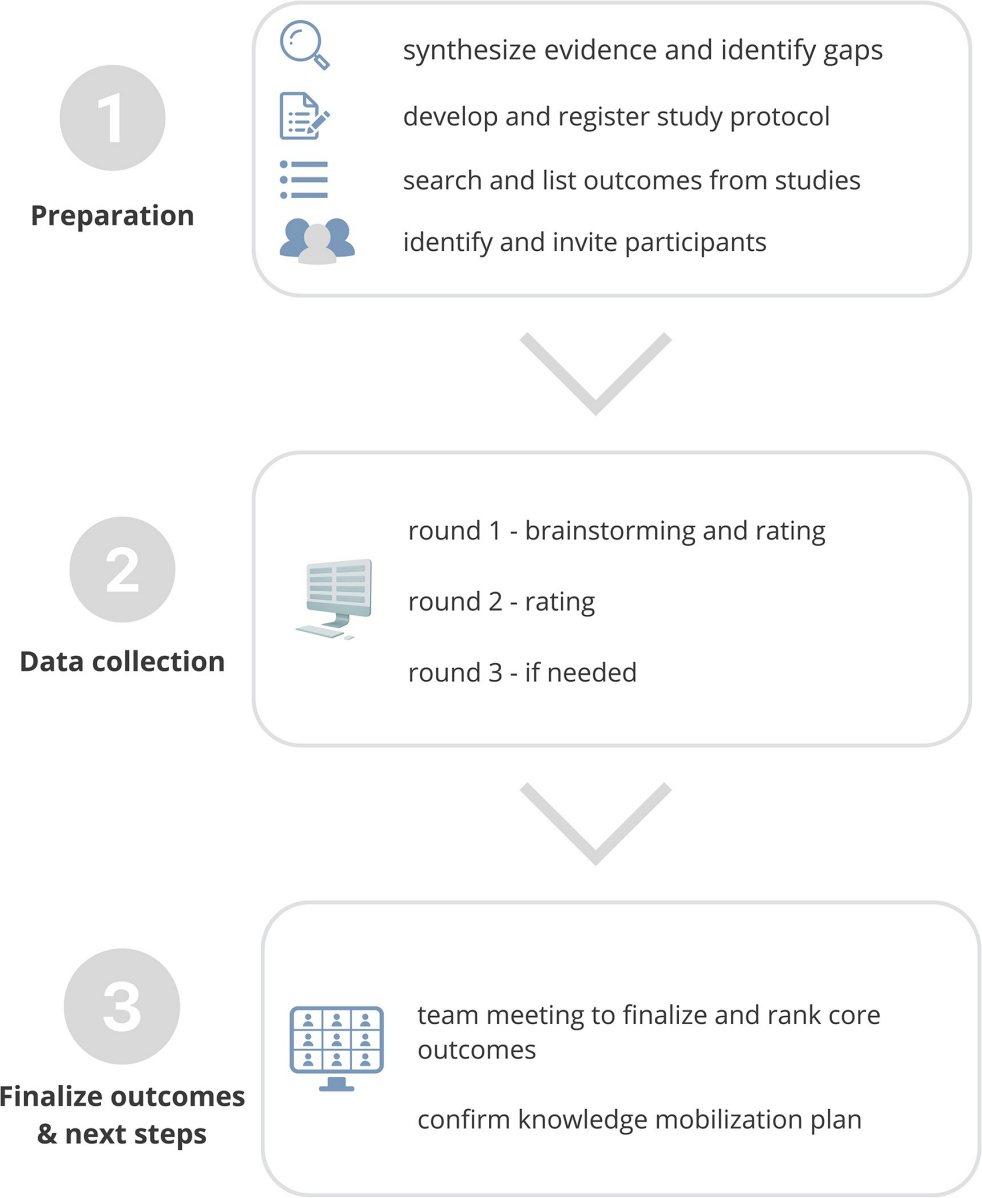
An overview of the process involved in identifying and finalizing core outcomes for social prescribing and knowledge mobilization strategies.

We used the Conducting and REporting DElphi Studies (CREDES) guideline to design this study [52]. Operationally, we will use methods based on the COMET Handbook [53], including, (1) reviewing evidence to identify types of outcomes for social prescribing (via a review of the published literature); (2) collecting feedback from online surveys to rate outcomes; and (3) holding a virtual team meeting to finalize the core outcome set. We modified the Delphi method by prioritizing the perspectives of middle-aged and older adults (as team members and survey participants) and using a virtual team meeting to finalize the outcomes within our large interdisciplinary team. Fig 1 is an overview of this study protocol and Fig 2 is an outline of the steps used within the Delphi method.

We obtained ethics approval from the Behavioral Research Ethics Board at The University of British Columbia (H22-03569); and we registered the protocol with the COMET database (https://www.comet-initiative.org/Studies/Details/2364).

### Composition and role of research team

**Fig 3:**
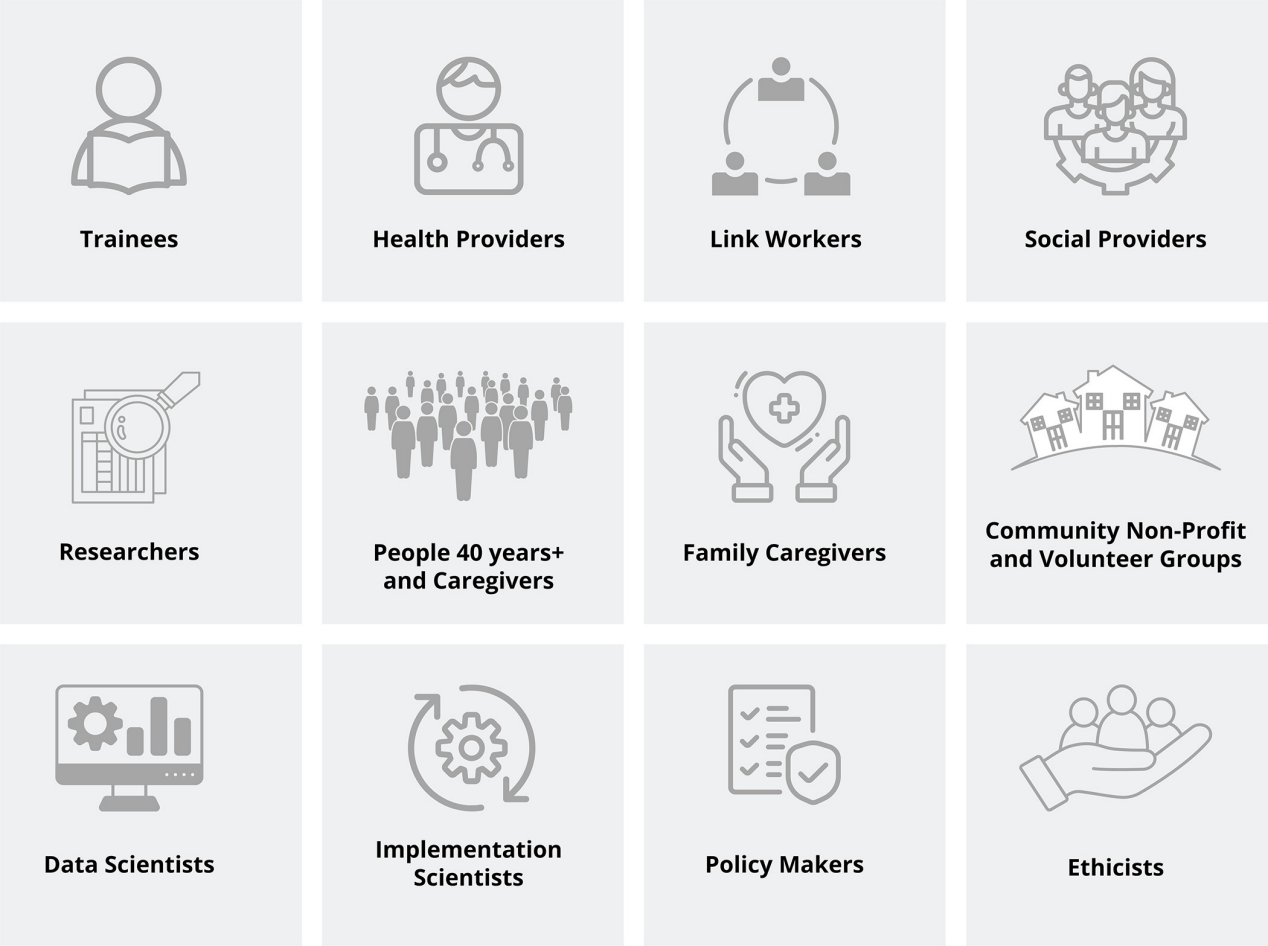
For our team composition and the consensus process, we will invite people from the following participant groups listed above. In this work, a link worker or community connector is a role held by people who support people to (re)engage with community resources or other opportunities based on identified needs.

The research team comprises people from some of the groups summarized in Fig 3, including people who receive social prescribing (or their caregivers), trainees, health and social providers, community-based organizations, and researchers. The International Association for Public Participation (IAP2)’s Public Participation Spectrum [54] guides how we conceptualize our approach to engaging diverse members of our interdisciplinary research team. Members will be invited to take part in all components of this study and will “collaborate” in decision making as defined by the IAP2 Spectrum [e.g., *“partner with the public in each aspect of the decision including the development of alternatives and the identification of the preferred solution*.” [54]]. Our engagement activities will include regular virtual team meetings aimed at planning and discussing the study’s progress across its research cycle and contributing to the development of study-related materials (e.g., patient-facing materials, knowledge mobilization products). An operations team (composed of trainees, and the first, second, and last author) formed from the larger group will closely monitor the study progress and evaluate its methodological aspects for consistency and rigor (e.g., via field notes and regular check-ins). An operations team member will also be appointed as a liaison to the knowledge users team members to ensure that they have a point person with any concerns and to uphold the integrity of the engagement process. We will establish and follow a terms of reference document co-developed by the team at the study’s outset [55]. Team members will participate in the larger online Delphi surveys (described below) but will be asked to self-identify as a team member within the pre-survey questions, so we can conduct a sensitivity analysis. We will present results separately for the research team and the larger group of invited participants. Finally, to ensure team members feel they can collaborate (as outlined by the IAP2 Spectrum) we will use a questionnaire, the Public and Patient Engagement Evaluation Tool [56], twice over the consensus study’s duration (mid-point and final) to learn from and evaluate our engagement process. We will also regularly check in with all team members to ensure they feel they can collaborate (as outlined by the IAP2 Spectrum).

### Part 1: Identifying types of outcomes used for social prescribing

We will identify potential outcomes from systematically reviewing the existing literature and feedback from our research team [23]. We recently published two systematic reviews on social prescribing and older adults [2, 57] and will review outcomes identified in studies within these syntheses. We will also identify other published systematic reviews of social prescribing studies via searching the following databases: MEDLINE and Embase (Ovid), EBSCO (APA Psych Articles, APA PsycInfo, CINAHL Complete, Social Work Abstracts, SPORTDiscus), Cochrane Database of Systematic Reviews, and Epistemonikos. One author (MCA) will use keywords “social prescribing” OR “social prescription” and filter the search with “review” to develop the search strategy. We will include reviews focussed on adults or older adults (without a specific health condition). We will include publications from all languages (using Google Translate, when able, to read the publication in English) and years. Two team members (EE and MCA) will independently review the titles, abstract and full texts to identify the reviews. Once systematic reviews are identified, both team members will independently review outcomes measured across studies. They will also extract the following information: author, publication year, systematic review question(s), population, setting, and outcome measures (instruments, such as the name of the questionnaire or performance-based measure).

For each outcome identified, we will categorized them into one of the six categories defined according to the taxonomy developed for outcomes in medical research [58]: (1) mortality/survival; (2) physiological/clinical; (3) functioning; (4) delivery of care; (5) resource use; and (6) adverse events/effects [58]. This approach differs from the core outcomes for social care previously noted, where the study categorized outcomes based on what was identified in the literature [47]. The two team members who extract the outcomes will categorize them using the taxonomy, and this list will be reviewed by two different team members (first independently then in a virtual discussion) to decide on the proposed synthesized list of outcomes. During the categorization process, it is also possible additional categories may emerge; or we need to reconsider the categorization of outcomes. In this case, two team members will discuss any items and present their suggestions to the team.

### Part 2. Online surveys to rate outcomes

*Survey Development and Piloting*: We will use DelphiManager (Software, DelphiManager, Core Outcome Measure for Effectiveness Trials Initiatives, Liverpool, UK) to design and administer survey rounds. We will provide participants access to the DelphiManager via email and assign each participant a unique number. For each survey question (on types of outcomes), we will ask participants to rate the importance of each outcome to social prescribing on a scale from one (which indicates “not important”) to nine (which indicates “essential”). Two authors will draft the first surveys and send them to all team members who will review and provide feedback in advance of starting the survey process.

#### Study participants

Selection of the participants is essential for conducting a robust Delphi study [51]. Participants should represent and/or have experience with the topic [20]; here, participants would be people "*affected by or who affect change”* within social prescribing [28]. In particular, for people receiving social prescribing we are focused on adults 40 years and older, when the perception of aging starts [48, 49], and their caregivers. Available evidence highlights variability in the size of the number of participants (i.e., the panel) which can range between four and 3,000 people [34]. The recommended number for panel membership is 10 to 18 people [52] to facilitate the consensus-building process [52]. Thus, we will engage a larger group of participants to brainstorm and provide feedback on core outcomes within the surveys. However, our team will be the panel who reviews and finalises the core outcome set.

Acceptance rates to participate in a Delphi study varies from about 25% [20] to 75% [59]. Although Junger and colleagues suggested a panel size of 10–18 was beneficial for group dynamics, we used this guidance to estimate a sample size for subgroups of participants who will brainstorm and rate outcomes for social prescribing. Specifically, we propose 12 subgroups of participants based on our previous framework for older adults’ community mobility [60] and listed in Fig 3. We will also include data scientists and ethicists as study participants to consider the role of data integration within social prescribing, and the importance of ethics in digital health [61], a possible delivery mode. Therefore, we aim to enroll up to 10 people for each of the 12 sub-groups of people (n = 120) [52]. However, we recognize we may not be able to enrol 10 data scientists or ethicists. Conservatively assuming a 50% rate of enrollment [20] we will invite 240 people to contribute to the Delphi process.

For our recruitment strategies, we will create a list of people with social prescribing experience (research, practice, or membership in a social prescribing organizations) through a number of methods. For example, we will (i) search related publications located in the systematic reviews identified in Part 1; (ii) approach social prescribing organizations to send out information on the project to their membership; (iii) identify providers experienced with social prescribing in Canada (via websites, related organisations, and publications); and (iv) via adults/older adults and/or caregivers who receive social prescribing in Canada [via posters and informational presentations (community engagement) at social prescribing sites in Canada]. Once identified, we will contact people via email and invite them to take part in the study. We will inform potential participants about the aim of the study, methods, confidentiality, and the time needed to complete surveys. We will limit information provided to potential participants, to reduce the risk of creating (unintentional) bias.

#### Strategies to engage participants

To improve the response rate, we will use different strategies, including: (i) providing a clear explanation (and/or creating infographics or a video) of the study process, timeline, and commitment throughout the process (e.g., a priori communication plan); (ii) being responsive to participants’ emails and feedback, and in particular, sending study details in a timely manner (within 2–3 business days); (iii) sending an email reminder at identified times if a response is not received at the anticipated date; and (iv) as mentioned previously, we will provide in-person presentations on social prescribing and the study (if possible). We will not provide an honorarium to participants.

#### Delphi survey rounds

*Round 1*: We will use an online (up to) three round Delphi method to brainstorm and rate the importance of outcomes in social prescribing. Participants will be asked to complete each survey round within two weeks. In the first-round, participants will ask to complete some background information for the team to better understand their perspectives. We will also ask them to self-select (if possible) from the list of groups provided in Fig 3. We will not ask participants to provide any identifying information. The list of outcomes will be presented randomly to participants within surveys order to reduce the risk of bias [62]. Participants will also have the opportunity to suggest additional items. Finally, there will be space for participants’ written feedback about the selection of rating scores.

We will summarize the number of respondents and scores for each outcome using frequencies and means. We will also calculate the frequency of participants scoring one to three, four to six, and seven to nine [23]. We will review data in text boxes to identify possible additional outcomes to include in the subsequent round; and to understand why people made their rating choices.

*Round 2*: We will invite the participants from the first round to take part in the second-round survey. We will present the first-round findings to participants including a summary of their responses and average scores for each question. We will ask participants to rate each outcome (listed in a random order), where no consensus was reached during the first round, within a two-week time period. We will summarize the number of respondents and scores for each outcome using frequencies and means. We will also calculate the frequency of participants scoring one to three, four to six, and seven to nine [23]. We will analyze findings between groups to clarify perspectives and responses.

*Round 3*: A third and final round survey may be sent to rate outcomes.

#### Guidance for developing agreement (consensus)

During the multiple rounds of surveys, we will categorize outcomes into one of three groups. We will reach consensus to include an outcome when at least 75% of participants rate an outcome between seven and nine (i.e., the outcome is “essential”) and fewer than 20% of participants rate the outcome as “not important” (rated between one and three). Similarly, we will reach consensus to exclude an outcome if more than 75% of participants indicate the outcome is “not important” and fewer than 20% of participants rate the outcome essential. If either of these two conditions are not met, consensus will not be met, and participants will be asked to review and possibly revise their ratings in light of the group scores.

### Part 3: Virtual team meeting to finalize the core outcome set

Following the identification of outcomes and survey parts of this study, we will hold a virtual meeting to permit the research team representing selected knowledge user groups (based on Fig 3) to finalize and rank the core set of outcome measures. The meeting is a key element of the modified Delphi method: it will permit the research team to provide further clarification and justify their viewpoints [63]. We will send the team a summary of the results prior to the meeting.

The operations team will start the meeting by providing a summary of the process and findings. The team will also discuss any issues or “non-consensus” items which rise arose within the Delphi process. We will discuss all “excluded” and “no consensus” outcomes from the Delphi process to ensure key outcomes have not been excluded from the set for policy decisions and intervention adoption (like cost-effectiveness outcomes) or which may be important to patients and caregivers. We will also review responses from subgroups to make sure there is consistency across knowledge user groups.

### External validation of core outcome set

It has been suggested an external panel or board review the results from the core outcome set to support the validity of findings. After finalizing the core set of outcomes, we will request posting the synthesized data on social prescribing related websites for a two-week period to seek feedback. We will also hold presentations in the community to seek feedback on the study and its findings.

### Knowledge mobilization

Our team includes a range of people and perspectives, and we adopt an integrated and participatory approach from the beginning. For our end of project knowledge mobilization, we will use a variety of strategies to engage different audiences, as guided by our interdisciplinary research team. We will publish a manuscript and submit conference abstracts. We will create a presentation for disseminating information and community engagement. We will also use this current synthesis process to inform our next research phase–to review the specific instruments (and their psychometric properties) used with social prescribing.

## Discussion

Social prescribing is becoming more prevalent within the literature and (publicly funded) health care systems. However, despite its potential to have a positive impact at multiple levels, there remain opportunities to integrate robust research approaches within its delivery and evaluation. Our previous evidence syntheses [2, 57] underscored the need to identify important and relevant outcome measures for social prescribing. Therefore, we will address this knowledge gap by using robust Delphi methods [53] to engage a large diverse group of people and organizations to co-create a core outcome set for social prescribing. This study will advance the field by taking a comprehensive and participatory approach to include perspectives from a wide range of knowledge users. We anticipate the findings of this study will provide researchers and knowledge users with outcomes at the person, provider, program, and societal-level for future studies.

### Potential limitations and strength of the proposed study

The main limitation with this work is the methodology. Although the Delphi method has many strengths, findings are based on consensus and reflect the perspectives of the people who contributed [64]. However, following the guidance from CREDES [52] we have included several steps to strengthen our work. For example, we registered the protocol with COMET; provided a detailed description of our methods (including additional components beyond the traditional Delphi method) in this protocol; defined the level of agreement required before starting the consensus process; and we will report the findings within the context of the methods (i.e., results are based on perspectives, report on topics which did not receive group consensus) [64].

We seek to be inclusive within team composition and invited participants. However, we recognize it is often difficult to recruit participants for research studies in general. As a result, findings will be guided by who takes part in the consensus process. However, we will also ask team members to provide feedback on how to identify people from hard-to-reach populations and how outcomes may vary by factors such as age, ethnicity, culture, gender, gender identity and expression, and sexual orientation; living in low resource communities; from small urban or rural settings; living with disabilities, and other factors which contribute to a person’s identity.

We will make every effort to recruit a consistent number of participants for each subgroup. However, based on our initial search of social prescribing researchers and organizations, we anticipate some challenges identifying data scientists and ethicists with social prescribing experience/knowledge. If needed, we will expand the inclusion criteria for underrepresented groups to participants with experience in middle-age/older adult health and social care.

We will review responses from subgroups to make sure there is consistency across knowledge user groups. The results may be affected by heterogeneity within and between participant groups. However, we intentionally adopted a modified Delphi method and will review the findings at the virtual meeting. Specifically, the team will discuss which items were excluded, and the distribution of data for subgroups and overall.

Further, the use of an online survey will limit the participation to people who are familiar with, and have access to, a computer and the internet. In addition, we will only include adults/older adults and/or caregivers who receive social prescribing in Canada. Although we will aim to recruit participants from distinct parts of Canada, the importance of outcomes may vary between locations and across settings.

### Conclusion

We provide a protocol for a comprehensive and participatory approach for the development of core outcomes for social prescribing. A key strength of this work is the inclusion of the focus on the perspectives of “*people who receive*” social prescribing and begin to shift the dynamic which often place people on the *“receiving end”* of care. As with most evidence synthesis, this work will be a living document, and findings will change as social prescribing evolves and develops for different settings and populations. However, our goal is to create a conversation and a foundation to ensure what is measured responds to the needs of people, communities, and society.
